# Peripheral and central neuroimmune mechanisms in Alzheimer’s disease pathogenesis

**DOI:** 10.1186/s13024-025-00812-5

**Published:** 2025-02-21

**Authors:** Shuo Zhang, Yue Gao, Yini Zhao, Timothy Y. Huang, Qiuyang Zheng, Xin Wang

**Affiliations:** 1 State Key Laboratory of Cellular Stress Biology, Fujian Provincial Key Laboratory of Neurodegenerative Disease and Aging Research, Institute of Neuroscience, Department of Neurology, School of Medicine, the First Affiliated Hospital of Xiamen University, Xiamen University, Xiamen, 361102 Fujian China; 2 Shenzhen Research Institute of Xiamen University, Shenzhen, 518057 Guangdong China; 3 Neuroscience Initiative, Sanford Burnham Prebys Medical Discovery Institute, La Jolla, CA 92037 USA

**Keywords:** Alzheimer’s disease, Peripheral immune cell, Microglia, Neuroimmune interaction, Blood-brain barrier

## Abstract

Alzheimer’s disease (AD) poses a growing global health challenge as populations age. Recent research highlights the crucial role of peripheral immunity in AD pathogenesis. This review explores how blood-brain barrier disruption allows peripheral immune cells to infiltrate the central nervous system (CNS), worsening neuroinflammation and disease progression. We examine recent findings on interactions between peripheral immune cells and CNS-resident microglia, forming a self-perpetuating inflammatory cycle leading to neuronal dysfunction. Moreover, this review emphasizes recent developments in the dysregulation of immune factors from both the periphery and CNS, and their impact on AD progression. With ongoing research and development of new therapeutic strategies, this review underscores the importance of modulating interactions between the peripheral immune system and CNS in AD therapy.

## Introduction

Alzheimer’s disease (AD), a major neurodegenerative disorder, presents a growing global challenge, with its prevalence increasing alongside an aging population [1]. While amyloid plaques and neurofibrillary tangles have traditionally been the focus of AD research, recent evidence highlights the critical role of peripheral immunity in AD pathogenesis, prompting a reevaluation of the disease’s underlying mechanisms [1–3]. The integrity of the blood-brain barrier (BBB) emerges as a crucial factor in AD progression, as its compromise facilitates the infiltration of peripheral immune cells into the central nervous system (CNS), exacerbating neuroinflammation and accelerating cognitive decline [4].

Emerging research elucidates the complex bidirectional communication between peripheral immune cells and CNS-resident microglia [5–8]. Various immune cells, including T cells, macrophages, natural killer cells, neutrophil, and B cells, modulate the neuroinflammatory milieu in the AD brain, contributing to a self-perpetuating inflammatory cascade [9]. The dysregulation of key immune mediators, such as pro-inflammatory cytokines, apolipoprotein E4 (APOE4), β2 microglobulin (B2M), and the microglial receptor TREM2, further complicates the neuroimmune landscape, influencing AD progression [9, 10]. This review discusses recent progress in understanding the role of the peripheral immune system in AD, with an emphasis on immune-CNS communication and potential therapeutic targets. It identifies promising areas for future research and explores novel therapeutic approaches for addressing neuroimmune interactions in AD.

## The function and mechanism of peripheral immune cells in AD

### CD8+ T cells

#### Infiltration of CD8+ T cells into the brain

The BBB is a highly selective structure formed by tightly connected endothelial cells of the brain microvasculature [11]. Its primary function is to protect the CNS from external substances and blood-borne immune cells, thereby maintaining CNS homeostasis [11, 12]. The BBB consists of specialized endothelial cells supported by pericytes and astrocytic end-feet, with its restrictive permeability ensuring that only molecules with specific receptors or transport proteins can traverse the endothelial layer [11, 12]. Current evidence from experimental models, including transgenic mouse models and *in vitro* BBB systems, suggests that this protective function becomes progressively compromised in Alzheimer’s disease-like conditions [13, 14]. While these findings provide important mechanistic insights, further validation in human AD brain samples would strengthen our understanding of BBB dysfunction in clinical settings. During disease progression, Aβ peptides not only aggregate as parenchymal plaques but also accumulate within the meningeal walls and cerebral vasculature, resulting in cerebral amyloid angiopathy (CAA) [15, 16]. This pathology further compromises BBB integrity, leading to vascular dysfunction, ischemia, microhemorrhage, and increased infiltration of peripheral immune cells [16, 17].

In AD patients, BBB dysfunction facilitates the infiltration of peripheral immune cells, particularly CD8^+^ T cells, into the CNS, thereby exacerbating neuroinflammation and accelerating disease progression [17]. While the exact mechanisms of CD8^+^ T cell entry into the brain parenchyma remain under investigation, current evidence suggests potential pathways through the dural lymphatics, choroid plexus, and cerebral blood vessels [18–20]. Studies have shown that the number of CD8^+^ T effector memory CD45RA (T_EMRA_) cells in the blood is increased and negatively correlates with cognitive function in AD patients [5]. Notably, clonally expanded CD8^+^ T_EMRA_ cells that possess Epstein-Barr virus (EBV)-specific T-cell receptors (TCRs) have been detected in the cerebrospinal fluid (CSF) of AD patients, as demonstrated by single-cell TCR sequencing analysis [5]. Infection with EBV has been proposed to increase the risk of developing AD [21]. Similar observations have been made in animal models, specifically in Tau transgenic mice, where clonal expansion of both CD8^+^ and CD4^+^ T cells has been observed [22].

The regulation of CD8^+^ T-cell transmigration and function, particularly in early AD, may be crucial for preventing further BBB deterioration and disease progression. Recent studies using a 3D human neuroimmune axis model have demonstrated that blocking CXCL10-CXCR3 interactions through anti-CXCR3 neutralizing antibodies markedly reduces the exacerbation of AD pathology caused by infiltrating CD8^+^ T cells [6]. While 3D culture systems provide valuable insights into the roles and effects of CD8^+^ T cells, they have inherent limitations in replicating the full complexity of the immune system. Most notably, these models cannot fully recapitulate critical physiological features, such as BBB functionality and meningeal immune cell dynamics. Given these constraints, integrating findings from 3D models with *in vivo* studies in animal models becomes essential for understanding CD8^+^ T cell mechanisms in a more physiologically relevant context. Similarly, co-administration of CCL2- and CCL8-neutralizing antibodies has been shown to attenuate immune cell penetration across the BBB [7]. Furthermore, blocking CCL3 can partially reduce CD8^+^ T-cell recruitment in aged brain [8]. Thus, effective management and inhibition of CD8^+^ T-cell infiltration and activity may mitigate pathological progression in AD.

#### Interaction between CD8+ T cells and microglia

**Fig. 1:**
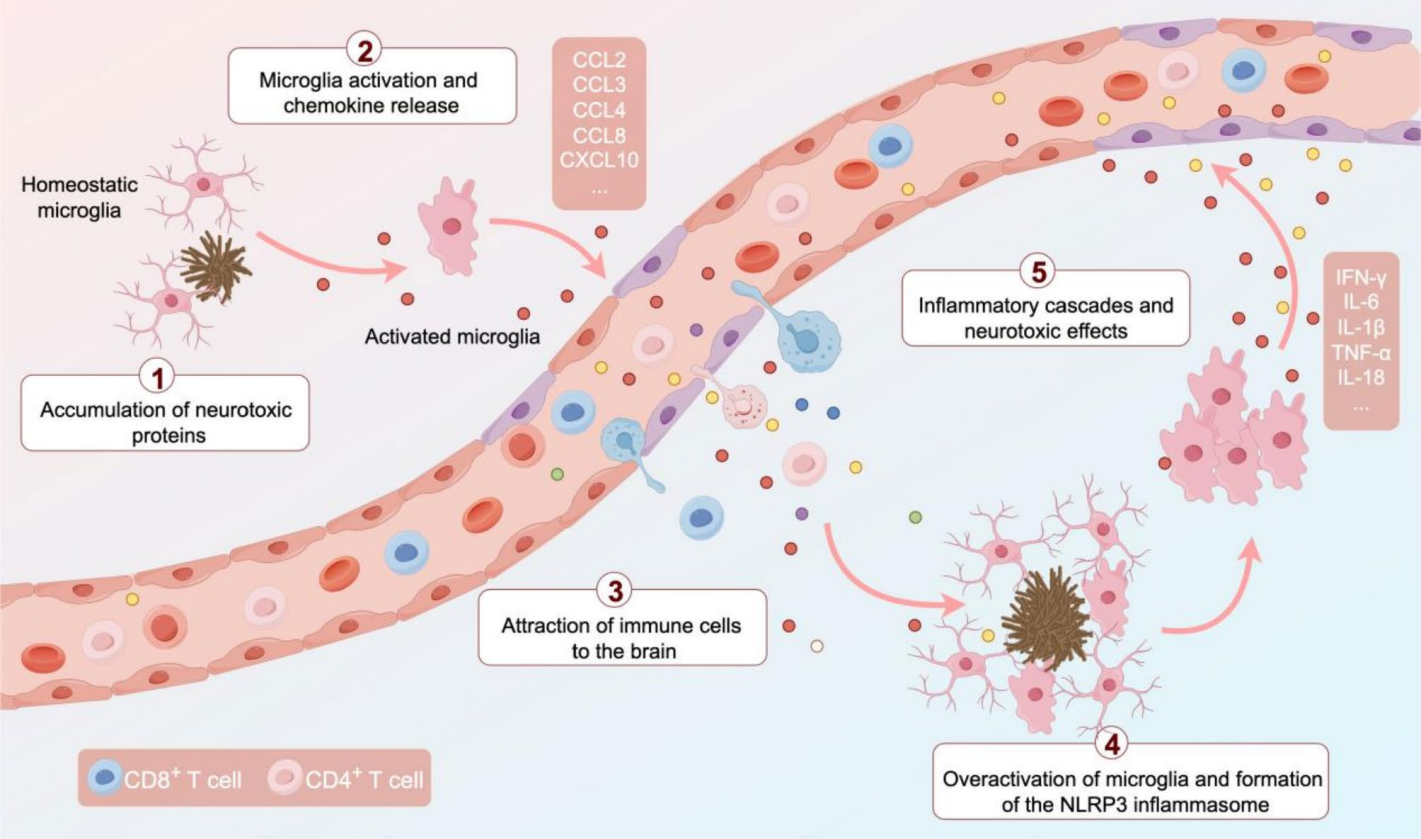
A self-sustaining feedback loop of neuroinflammation mediated by the interplay between T cells and microglia. Neurotoxic protein (Aβ and tau) accumulation in AD triggers microglial activation, initiating a cascade of pathological events. Activated microglia release chemokines that recruit T cells to the brain parenchyma, where T cells secrete cytotoxic factors that exacerbate Aβ production and aggregation. This further amplifies microglial activation, leading to the assembly of the NOD-, LRR-, and pyrin domain-containing protein 3 (NLRP3) inflammasome. This critical multi-protein complex activates caspase-1, driving the release of pro-inflammatory cytokines, including IL-1β and IL-18. The resulting self-sustaining cycle intensifies T-cell infiltration and neuroinflammation, ultimately accelerating neurodegeneration.

In AD brain, microglia are highly responsive to deposition of neurotoxic proteins such as amyloid-β (Aβ) and tau. Upon activation, microglia release chemokines that attract CD8^+^ T cells to facilitate their infiltration into brain tissue [7, 22]. Infiltrating CD8^+^ T cells secrete cytotoxic factors, including perforin and granzymes, which further potentiate microglial activation, resulting in increased production of pro-inflammatory cytokines, including interferon-γ (IFN-γ), interleukin-1β (IL-1β), and tumor necrosis factor-α (TNF-α) [3, 5, 23]. These cytokines not only propagate the inflammatory milieu, but also augment Aβ production and aggregation while impeding Aβ clearance [24–26]. These events exacerbate BBB dysfunction, and promote further infiltration of CD8^+^ T cells into brain tissue. This interaction establishes a self-perpetuating cycle, escalating CD8^+^ T-cell infiltration and neuroinflammatory responses, thereby accelerating neurodegenerative progression (Fig. 1).

A significant increase in CD8^+^ T cells within regions enriched for tau pathology has been observed in both tauopathy mouse models and AD human brain [27]. Microglia-mediated T-cell infiltration leads to neuronal dysfunction and death, disruption of neuronal communication and memory formation. In aging brain, the CD8^+^ T-cell-initiated inflammatory cascade serves as a critical driver of neurodegenerative processes [28]. CD8^+^ cytotoxic T cells are a primary source of IFN-γ, which induces IFN-responsive microglia, thereby triggering neuroinflammatory events in the CNS [28]. IFN-γ exerts direct effects on oligodendrocytes, microglia, and neural stem cells, suggesting that CD8^+^ T cells contribute to oligodendrocyte death and axonal degeneration, leading to cognitive impairment and decline in motor function [28]. In the 5×FAD mouse brain, CD8^+^ T cells exhibit close spatial association with microglia in the vicinity of amyloid plaques, with their recruitment being mediated by peripheral B cells [29]. Therapeutic strategies targeting microglia-CD8^+^ T-cell interactions, such as microglial or T-cell depletion or the inhibition of IFN-γ signaling, may mitigate tau-mediated neurodegeneration and attenuate AD-associated neuroinflammation and neurodegenerative changes [22, 27].

### CD4+ T cells

CD4^+^ T cells exhibit remarkable plasticity, capable of differentiating into distinct effector subsets in response to specific cytokine environments [30]. These subsets can be broadly categorized into pro-inflammatory and anti-inflammatory phenotypes, each playing distinct roles in AD pathogenesis [31]. The pro-inflammatory subsets, primarily T helper type 1 (Th1) and Th17 cells, contribute to BBB disruption and enhanced microglial activation, thereby exacerbating AD-associated neuropathology [32, 33]. In contrast, anti-inflammatory subsets, including Th2 cells and regulatory T cells (Tregs), exhibit neuroprotective properties and can attenuate neuroinflammation [34, 35]. This functional dichotomy of CD4^+^ T-cell responses underscores the intricate immunological landscape in AD and suggests potential targets for intervention.

#### A pro-inflammatory role for CD4+ T cells

Th1 cells activate microglia through secretion of IFN-γ to promote clearance of amyloid plaques in APP/PS1 mouse brain [32]. However, hyperactivated Th1 cells can produce excessive levels of IFN-γ and TNF-α, and result in Aβ accumulation and microglial overactivation, thereby exacerbating neuroinflammation and cognitive deficits [32, 33]. In APP/PS1 mice, adoptive transfer of Aβ-stimulated Th1 cells increases cerebral Aβ burden and impairs synaptic plasticity [34]. Limited therapeutic efficacy of anti-Aβ monoclonal antibodies and Aβ vaccines may be attributed to their propensity to induce Th1 cell-mediated inflammatory responses, potentially leading to excessive immune activation [36].

Th17 cells demonstrate robust brain infiltration and produce a variety of cytokines, such as IL-17 A, IL-23, IL-21, IL-6, and IFN-γ, which trigger neuroinflammatory responses characterized by microglial hyperactivation and recruitment of additional immune cells [33, 37]. In APP/PS1 transgenic mice, adoptive transfer of Aβ-specific Th1 and Th17 cells, which recognize Aβ through specific TCRs, accelerates memory impairment and elevates TNF-α, IFN-γ, and IL-17 levels in the blood [33]. Moreover, IL-17 contributes to AD progression by enhancing neuroinflammation, inhibiting microglial phagocytosis, and aggravating amyloid deposition. Conversely, IL-17 neutralization can mitigate cognitive impairment and synaptic dysfunction [37, 38].

**Fig. 2:**
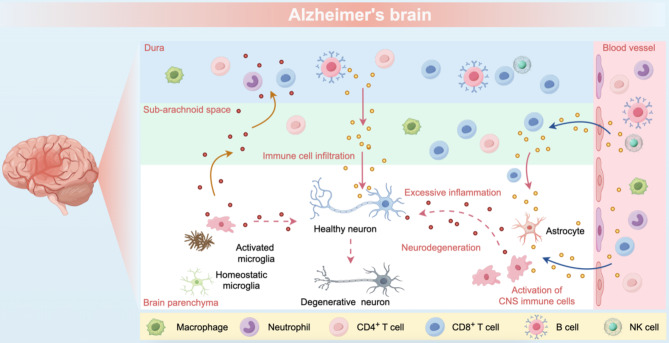
Impact of peripheral immunity on the CNS in AD brain. In AD, peripheral immune cells infiltrate the dura mater, subarachnoid space, and brain parenchyma, disrupting CNS immune homeostasis through clonal expansion, abnormal antigen accumulation, and excessive inflammatory mediator release. Within the brain parenchyma, microglia and astrocytes transition from homeostatic to reactive states, amplifying inflammation and accelerating neurodegeneration. This cascade of events progressively compromises neuronal integrity, leading to structural and functional deterioration.

T helper type 22 (Th22) cells are characterized by their production of IL-22 without co-expression of IL-17 or IFN-γ [39]. In AD brain, elevated IL-22 levels activate glial cells, leading to pro-inflammatory cytokine production and lymphocyte infiltration into the brain parenchyma [40]. Concurrently, T helper type 9 (Th9) cells are markedly upregulated in AD, resulting in enhanced IL-9 production [40]. IL-9, in combination with TGF-β1, facilitates the differentiation of naive CD4^+^ T cells into Th17 cells, which are also elevated in the peripheral blood mononuclear cells of AD patients [40, 41] (Fig. 2).

#### An anti-inflammatory role for CD4+ T cells

Th2 cells stimulated by Aβ suppress IFN-γ production in Th1 and Th17 cells, thereby downregulating CD86 and CD40 expression in microglia and attenuating subsequent pro-inflammatory responses [34]. In APP/PS1 mice, administration of purified Th2 cells reduces IFN-γ, TNF-α, GM-CSF, IL-2, IL-4, and Aβ levels in plasma, while enhancing cognitive function and diminishing plaque-associated microglia and vascular amyloidosis [42].

Tregs play a crucial role in controlling immune responses by suppressing the activation and proliferation of effector T cells through the secretion of anti-inflammatory cytokines, such as IL-10 and TGF-β [35]. Postmortem analyses of human AD brains have revealed a reduction in Treg populations compared to non-AD controls [43]. This reduction is associated with excessive production of pro-inflammatory cytokines such as TNF-α, IL-1β, and IL-6, leading to enhanced neuroinflammatory effects [43]. Furthermore, studies in APP/PS1 mice have shown that the transfer of Aβ-specific Th1 and Th17 cells diminished both the number and functionality of Tregs in the CNS and peripheral blood, further exacerbating neuroinflammation [33].

In APP/PS1 mice, peripheral administration of human IL-2 at low doses selectively induces the expansion of Tregs, restores the Treg/Th17 balance, increases plaque-associated microglia, and reduces amyloid plaques and neuroinflammation, thereby enhancing cognitive function [44]. These positive outcomes have led to the progression of low-dose IL-2 therapy to a phase 2 clinical trial (NCT06096090) for AD treatment [45]. Another potential treatment strategy involves direct Treg transfer. Autonomous ex vivo expansion and transfer of Tregs following Aβ stimulation have been shown to reduce amyloid load and microglial overactivation in 5×FAD mice, while downregulating pro-inflammatory cytokines and the complement cascade, thereby ameliorating cognitive impairment, Aβ accumulation, tau hyperphosphorylation, and neuroinflammation [46]. Additionally, Aβ-stimulated Tregs suppress pro-inflammatory microglial activity through bystander suppression [46].

However, contrasting evidence suggests that temporary depletion of FOXP3^+^ Tregs can also have beneficial effects. This depletion triggers an IFN-γ-dependent systemic immune response and activates the choroid plexus. Consequently, this activation leads to the infiltration of monocyte-derived macrophages (MDMs) and Tregs into the vulnerable brain region of amyloid pathology, resulting in a reduction of amyloid plaque burden and improved cognitive function in 5×FAD mice [47].

These findings highlight the complex dynamics of Treg modulation in AD. While enhancing Treg function might be beneficial in some contexts, their depletion could prove advantageous in others. This complexity underscores the need for tailored therapeutic strategies that consider the specific pathological context and immune environment in AD. As such, molecular targeting approaches that aim to enhance Treg inhibitory functions through pathways like TGF-β and IL-10 signaling are still under exploration [35, 48].

### Other peripheral immune cells

Macrophages, including border-associated macrophages (BAMs) and monocyte-derived macrophages (MDMs), play crucial roles in AD pathogenesis [49–52]. BAMs, residing in cerebral border regions, are vital for maintaining brain homeostasis by facilitating glymphatic system clearance [50–53]. However, age-related alterations in BAMs, such as enhanced major histocompatibility complex class II (MHC-II) expression and impaired extracellular matrix degradation, contribute to vascular and glymphatic dysfunction, thereby increasing the risk of AD [52, 54, 55]. In AD mouse models with amyloid pathology, BAM depletion exacerbates Aβ deposition and microglial activation [50].

MDMs accumulate with age and participate in Aβ clearance [56, 57], however, their capacity to take up Aβ diminishes with age and AD progression, partially due to decreased expression of phagocytic receptors, such as TREM2 [56]. Genetic variations in *TREM2* are associated with an increased risk of AD onset [58]. TREM2^+^ macrophages cluster around amyloid plaques, while TREM2-deficient mice exhibit reduced inflammation and attenuated pathology [59]. In TREM2-deficient AD mouse models, the number of CD45^hi^Ly6C^+^ macrophages is substantially decreased, resulting in attenuated inflammation and ameliorated amyloid and tau pathology [60].

Targeting the immune checkpoint blockade by anti-PD-1 antibodies results in the recruitment of MDMs to the brain, which subsequently facilitates the clearance of amyloid plaques and restores cognitive function in 5×FAD mice [61]. Notably, the effects of PD-1/PD-L1 blockade extend beyond MDMs, as it also disrupts negative signaling between IFNγ-producing T cells and their target cells, resulting in broad enhancement of immune responses [61]. Building on this approach, IBC-Ab002, a humanized IgG1 antibody that inhibits the related immune checkpoint protein PD-L1, has now entered phase 1 clinical trials (NCT05551741) [45].

Transcriptomic analyses and immunohistochemical characterization of postmortem human hippocampal tissue revealed that monocyte-derived macrophages infiltrate the brain parenchyma in late-stage AD [62] (Fig. 2). Nonetheless, replacing brain-resident microglia with peripheral monocytes does not affect the amyloid plaque load in AD mouse models with amyloid pathology [63, 64]. This finding prompts inquiries regarding the distinct functions of peripheral monocytes and microglia in the clearance of Aβ.

Natural killer cells, key components of the innate immune system, play complex and seemingly paradoxical roles in AD pathogenesis. Depletion of NK cells in the 3×Tg-AD mouse model has neuroprotective effects, including attenuated neuroinflammation, enhanced neurogenesis, and improved cognitive function [65]. Conversely, augmentation of NK cell function in the APP/PS1 mouse model can mitigate cerebral Aβ deposition and enhance cognitive performance [66]. These contrasting findings underscore the multifaceted nature of NK cells in AD. Recent clinical investigations, notably the ASK-AD trial (NCT04678453), have explored the therapeutic potential of autologous NK cell therapy (SNK01) in AD patients. Preliminary results indicate that SNK01 treatment may lead to reductions in cerebral Aβ and tau protein levels, coupled with amelioration of neuroinflammation.

Neutrophils play a pivotal role in cerebral hypoperfusion, a well-established phenomenon in AD, by adhering to capillary segments, which leads to reduced and eventually obstructed cerebral blood flow (CBF) [67]. Additionally, neutrophils compromise BBB integrity by disrupting tight junction proteins such as occludin and claudins, thereby promoting increased BBB permeability [68]. In transgenic AD models, such as 5×FAD and 3×Tg-AD mice, neutrophils extravasate and accumulate in regions of amyloid deposition, accompanied by the formation of neutrophil extracellular traps (NETs) and expression of IL-17 [69]. Aβ42 peptide induces a high-affinity conformation of lymphocyte function-associated antigen 1 (LFA-1), promoting rapid neutrophil adhesion to integrin ligands, which subsequently exacerbates AD pathology and cognitive decline [69]. Therapeutic approaches targeting neutrophils have shown promising results. Transient neutrophil depletion during early disease stages results in sustained cognitive improvements, while administration of Ly6G antibodies or LFA-1 inhibitors enhances cerebral blood flow, alleviate AD-like neuropathology, and improve cognitive function [67, 69]. Moreover, LFA-1-deficient transgenic AD mice demonstrate resistance to cognitive deterioration and exhibit reduced gliosis [69]. LFA-1 is not only crucial for NK cell responses but is also expressed by other peripheral immune cells, including T cells, where it plays essential roles in activation, adhesion, and migration [68–70]. The involvement of LFA-1 in these diverse immune cell functions suggests its potential significance in AD pathology and warrants further investigation.

B cells in AD patients became activated and accumulate in the periphery and brain parenchyma, where they produce immunoglobulins that target Aβ [70]. While B-cell-derived immunoglobulins that target Aβ may impede plaque formation and disease progression, they may concurrently impair microglial function and exacerbate AD pathology [70]. The role of B cells in AD extends beyond immunoglobulin production, as evidenced by the correlation between increased numbers of activated B cells in cervical lymph nodes from aged 3×Tg AD mice, which rise as the disease progresses [71]. B-cell depletion in 5×FAD and APP/PS1 mice is associated with attenuated disease progression, amelioration of cognitive and motor deficits, and a reduction in amyloid burden [71]. Paradoxically, B-cell depletion during the early disease stages accelerates cognitive decline and increases amyloid burden in AD mouse models, highlighting the complex and stage-dependent role of B cells in AD pathogenesis [72, 73].

Mucosal-associated invariant T (MAIT) cells, a subset of innate-like T cells, recognize microbial-derived metabolites, particularly those derived from vitamin B, through the major histocompatibility complex class I (MHC-I)-related protein MR1 [74]. In the 5×FAD mouse model, MAIT cell numbers progressively increase and display activation signatures [75]. Both AD patients and 5×FAD mice exhibit significantly elevated MR1 expression in microglia surrounding amyloid plaques [76]. Notably, amyloid plaque formation is markedly reduced in MR1-deficient mice [76].

Gamma delta T (γδT) cells, another subset of innate immune cells, serve as the primary source of interleukin-17 A (IL-17) in healthy meninges, where they support synaptic plasticity in CA1 hippocampal neurons and facilitate short-term memory [77]. However, in the 3×Tg AD mouse model, γδT cells accumulate substantially in the brain parenchyma and meninges, correlating with cognitive deterioration [37]. Notably, IL-17-producing γδT cells show marked increases in the CNS of these mice, with their accumulation associated with early short-term memory deficits and synaptic dysfunction [37]. These findings suggest that γδT cells may exacerbate neuroinflammation and contribute to the early AD pathological progression.

## Dysregulation of immune factors in AD

**Fig. 3:**
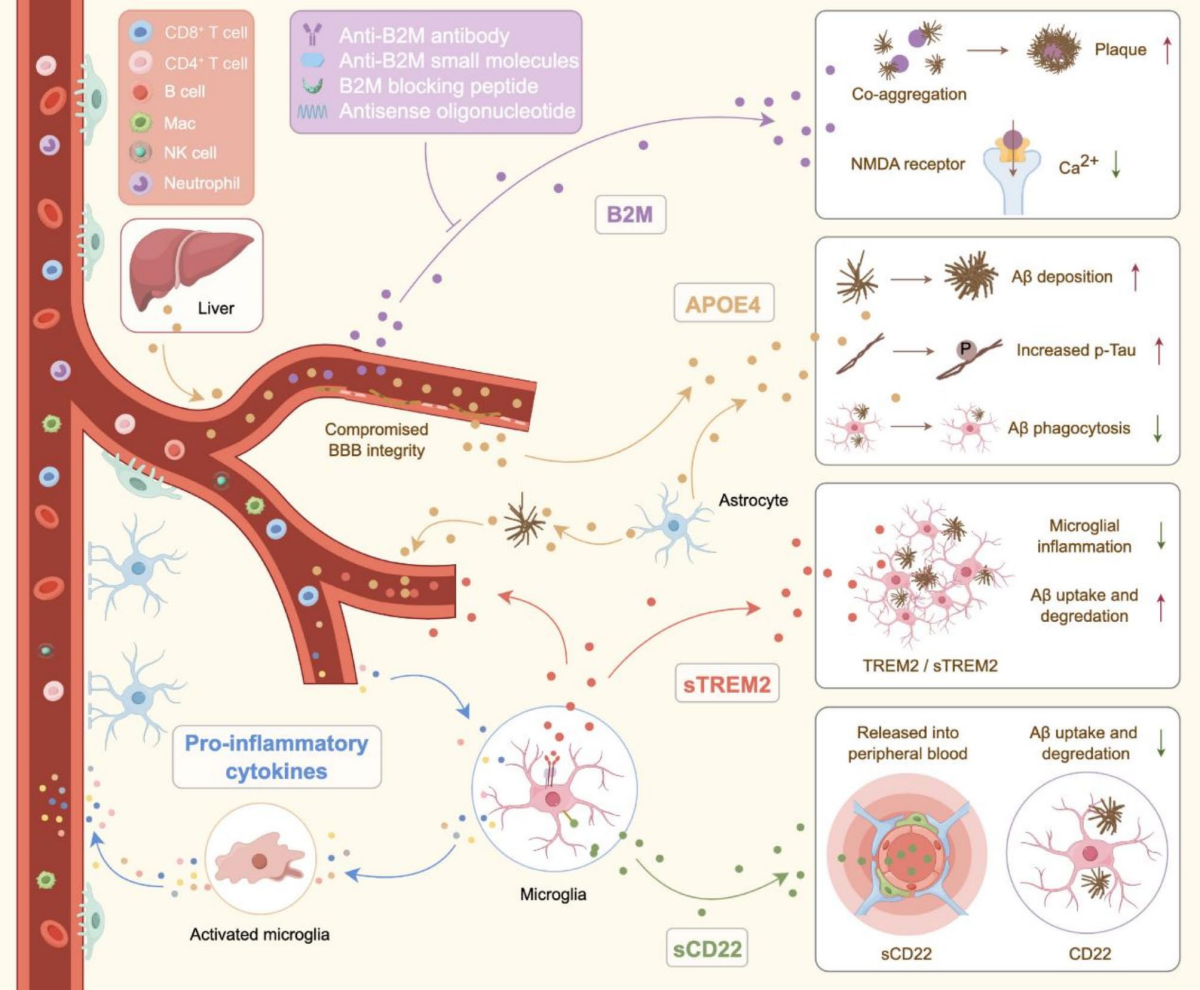
Dysregulation of immune factors in AD pathogenesis. In AD, compromised BBB integrity facilitates the entry of multiple immune factors, including pro-inflammatory cytokines, APOE4, B2M, TREM2, and CD22, into the CNS. These molecules play crucial roles in modulating neuroinflammation, maintaining BBB integrity, and regulating Aβ uptake and degradation. Elucidating the mechanisms associated with the dysregulation of immune factors in AD is essential for developing targeted immunomodulatory therapies.

Dysregulation of immune factors plays a pivotal role in AD pathophysiology and significantly affects the neuroimmune axis. Aberrant neuroimmune regulation not only facilitates peripheral immune cell infiltration into the CNS by compromising BBB integrity, but also exacerbates neurodegenerative processes directly [78]. Key neuroimmunological mediators implicated include pro-inflammatory cytokines, APOE4, B2M, TREM2, and CD22. These factors are instrumental in modulating neuroinflammatory responses and maintaining homeostatic immune interactions between the CNS and the periphery. Perturbation of these neuroimmunoregulatory factors intensifies neuroinflammation, compromises BBB function, and accelerates neurodegeneration through augmented peripheral immune cell infiltration. Consequently, elucidating the mechanisms underlying immune factor dysregulation and developing targeted immunomodulatory interventions are crucial for advancing novel therapeutic strategies aimed at attenuating and/or halting the progression of neurodegenerative disorders (Fig. 3).

### Pro-inflammatory cytokines

Pro-inflammatory cytokines, including interleukins (ILs), tumor necrosis factor (TNF) and interferons (IFNs), are predominantly secreted by peripheral immune cells [78]. In AD patients, these cytokines are markedly elevated in the plasma, facilitating their redistribution across a leakage-prone BBB associated with neurodegenerative progression [78].

Interferons are classified into Type I (IFN-I) and Type II (IFN-II, specifically IFN-γ) subtypes. IFN-I induces microglial activation and synaptic pruning, whereas selective inhibition of interferon α/β receptor (IFNAR) through specific blockers or neutralizing antibodies mitigates microgliosis and prevents synaptic loss in 5×FAD mouse model [79, 80]. IFN-II exhibits a biphasic role in neurodegenerative diseases. At physiological levels, IFN-γ enhances microglial phagocytosis, promotes Aβ clearance, and augments cognitive functions [81]. Additionally, IFN-γ-mediated immune responses recruit monocyte-derived macrophages to the CNS, facilitating amyloid plaque clearance and cognitive improvement [81]. However, chronic IFN-γ-induced inflammation compromises BBB integrity and allows peripheral inflammatory mediators to infiltrate the CNS, thereby exacerbating neuroinflammation, neuronal damage and cognitive decline [81, 82].

The pro-inflammatory milieu induces the upregulation and activation of NLRP3, forming the NLRP3 inflammasome and resulting in substantial production of pro-inflammatory cytokines [83]. In AD, NLRP3 inflammasome activation occurs predominantly in innate immune cells, particularly microglia in the CNS [83]. Hyperactivation of the inflammasome triggers an inflammatory cascade that exacerbates neuronal injury. Studies using 16-month-old APP/PS1 transgenic mice demonstrated that *Nlrp3* deficiency markedly enhanced microglia-mediated Aβ phagocytosis, reduced amyloid plaques, and improved spatial memory [84]. Furthermore, in transgenic Tau22 mice, *Nlrp3* deficiency attenuated tau hyperphosphorylation and aggregation while ameliorating Aβ-induced tau pathology [85].

### B2M

B2M, a key component of MHC-I complex, exists in circulation as a non-covalently associated monomeric protein that is predominantly cleared through renal glomerular filtration [86]. Under pathological conditions, such as chronic kidney disease with long-term dialysis, B2M forms amyloid fibrils, leading to amyloidosis and related disorders [86]. Recent research has highlighted the significant involvement of B2M in AD and Down syndrome (DS), where it affects cognitive function through both central and peripheral pathways [10, 87].

In AD, elevated B2M levels promote amyloid pathology through co-aggregation with Aβ, facilitating plaque formation and propagation [10]. This aggregation process enhances neurotoxicity and neuroinflammation, thereby accelerating both neurodegeneration and the spatial expansion of Aβ pathology [10]. Experimental studies in AD mouse models have demonstrated that B2M reduction—achieved through genetic ablation, antisense oligonucleotide (ASO) administration, or antibody-based interventions—markedly attenuates cognitive deficits and reduces amyloid plaque burden [10]. Additionally, the ability of B2M to traverse the BBB presents a promising therapeutic opportunity [10]. Notably, peripheral B2M clearance via systemic administration of low-dose anti-B2M antibodies effectively reduces brain B2M levels and improves cognitive function in AD mouse models, offering a potential alternative to high-dose Aβ antibody therapies, which frequently induce adverse effects [10].

Individuals with DS frequently develop AD by middle age, suggesting shared pathogenic mechanisms between these conditions [88]. In DS, elevated plasma B2M crosses the BBB and suppresses N-methyl-D-aspartate (NMDA) receptor activity, disrupting synaptic excitatory/inhibitory homeostasis and precipitating cognitive dysfunction [87]. Mechanistic studies have revealed that B2M directly interacts with the GluN1 subunit of NMDA receptors, attenuating receptor function. The development of GluN1-P2, a therapeutic peptide that competitively inhibits this B2M-GluN1 interaction, has demonstrated significant improvements in synaptic plasticity and cognitive performance in both DS and aging mouse models [87]. Through parabiosis studies and plasma transfer experiments, elevated circulating B2M has been identified as a critical mediator of cognitive impairment in both DS patients and mouse models [87]. Therapeutic interventions targeting peripheral B2M, including antibody neutralization and parabiotic circulation exchange, successfully reverse these cognitive deficits.

As a systemic aging factor, B2M levels increase with age in the plasma and CSF, contributing to cognitive decline in aging and neurodegenerative disorders [89]. The co-aggregation of B2M with Aβ enhances neurotoxicity, necessitating a refinement of the amyloid cascade hypothesis. Furthermore, B2M’s pathological interaction with NMDA receptors establishes it as a promising therapeutic target. These mechanistic insights provide a foundation for developing small-molecule inhibitors and antibody-based therapeutics targeting B2M-mediated pathology [10, 87].

### APOE4

The APOE4 gene variant encoded on human chromosome 19, is the most important genetic risk factor for late-onset AD [90]. APOE4 is characterized by arginine residues at positions 112 and 158 (Arg112/Arg158), distinguishing it from APOE3 (Cys112/Arg158) and APOE2 (Cys112/Cys158) [90]. APOE4 impacts both the CNS and peripheral immunity. In cognitively normal APOE4 carriers, the presence of plasma proteins in brain tissue and CSF suggests the presence of compromised BBB integrity prior to cognitive decline [91]. APOE4 potentially weakens endothelial tight junctions, increases BBB permeability and facilitates neuroinflammation [92], and enhances BBB breakdown by reducing the capillary basement membrane area and increasing thrombin levels in vessel walls and perivascular spaces [93, 94]. APOE4 also affects pathogenic changes in cerebral vasculature by promoting Aβ deposition and exacerbating CAA, further compromising BBB integrity and AD neuropathology [13]. Moreover, APOE4 impairs BBB-mediated Aβ clearance, shifting Aβ40/42 clearance from rapid clearance pathways mediated by low-density lipoprotein receptor-related protein 1 (LRP1) to slower clearance pathways mediated by the very low-density lipoprotein (VLDL) receptor pathway [95]. Conversely, APOE2 and APOE3 facilitate faster Aβ clearance at the BBB [95]. CAA progression alters Aβ clearance mechanisms and vascular pathology, including increased vascular pulsatility and reduced vascular smooth muscle cell coverage [13]. APOE4 also attenuates Aβ clearance by promoting premature meningeal lymphatic vessel atrophy [96].

APOE4 accelerates immunosenescence in neutrophils, resulting in the infiltration of immunosuppressive IL-17-producing neutrophils in the brains of female APOE4 carriers with AD. In human *APOE4* knock-in APP/PS1 mice, genetic ablation of *APOE4* in neutrophils diminishes their immunosuppressive phenotype and reduces IL-17 signaling. This alteration restores the microglial response, alleviates amyloid pathology, and enhances cognitive function in these AD transgenic mice [97]. Studies using mouse models expressing human APOE3 or APOE4 in the liver have demonstrated that liver-expressed APOE4 exacerbates cerebral amyloid pathology, whereas APOE3 reduces it. These findings suggest that peripheral APOE4 impairs cerebrovascular function, affecting synaptic plasticity and cognition, thus providing a rationale for targeting peripheral APOE4 in AD therapy [92]. Therapeutic strategies targeting APOE4, such as angiotensin receptor blockers, vascular endothelial growth factor A (VEGFA), and epidermal growth factor (EGF), can potentially improve BBB integrity and function and slow AD progression [98].

APOE is primarily synthesized and secreted by astrocytes and activated microglia in the CNS [90, 99]. In the PS19-APOE4 mouse model, astrocyte-specific APOE4 deletion reduces disease-associated gene expression across multiple cell types and suppresses aberrant synaptic phagocytosis [100, 101]. Neuron-specific APOE4 deletion markedly reduces tau pathology and ameliorates neurodegeneration [102], while microglial APOE4 deletion enhances Aβ clearance in APP/PS1 mice [103]. These cell type-specific effects reveal distinct contributions of APOE4 to AD pathogenesis.

### TREM2

TREM2, a member of the immunoglobulin receptor superfamily, is predominantly expressed in osteoclasts, macrophages, and microglia [104]. It activates downstream signaling pathways, including Ca^2+^ mobilization, MAPK, and mTOR, by binding to various ligands, such as phospholipids, APOE, Aβ oligomers, and apoptotic neurons [105–107]. In mouse models of AD, TREM2 exhibits neuroprotective effects primarily through its expression in microglia, where it serves as a critical regulator of immune responses in neurodegenerative diseases [108]. Studies in AD mouse models have also shown that *Trem2* knockout (*Trem2*^−/−^) or the presence of the TREM2 R47H mutation results in reduced plaque-associated microglia and increased dystrophic neurites [109]. Microglial reduction exacerbates Aβ toxicity and tau seeding and spreading [110]. Conversely, TREM2-activating antibodies enhance microglial metabolism and function, which leads to reduced amyloid plaque formation [111].

TREM2 undergoes ectodomain shedding at histidine 157 by α-secretases ADAM10 and ADAM17, generating soluble TREM2 (sTREM2) [112–114]. sTREM2 promotes microglial survival and plaque-associated clustering while enhancing both Aβ clearance and cytokine production [115]. Both TREM2 and sTREM2 significantly affect BBB function [116, 117]. Loss of TREM2 function or mutation can exacerbate neuroinflammation and neurodegenerative progression by increasing BBB permeability, thereby allowing peripheral immune cells and other inflammatory factors to enter the CNS [118].

As a potential link between the CNS and peripheral immune system, changes in sTREM2 levels can reflect the inflammatory state and severity of neurodegenerative changes within the CNS [119]. In AD patients, elevated sTREM2 levels in CSF and plasma are associated with early disease stages and mild cognitive impairment [120]. Increased sTREM2 correlates with memory decline and hippocampal atrophy [121]. Additionally, sTREM2 levels positively correlate with CSF tau and p-tau levels, indicating its potential applicability as a biomarker for AD progression [122].

### CD22

CD22, which was originally characterized as a B-cell receptor, has been found to exert important regulatory effects on microglia within the CNS [123]. Microglia maintain CNS homeostasis through phagocytic clearance of protein aggregates and cellular debris; however, this function deteriorates with age and in various neurodegenerative disorders associated with cognitive decline [123]. Studies have demonstrated that CD22 functions as a negative regulator of microglial phagocytosis and is upregulated in aged microglia [123]. CD22 is associated with exacerbated neuroinflammation and neurodegenerative changes through its anti-phagocytic effects derived from CD22 interactions with α2,6-linked sialic acid, which inhibits microglial phagocytosis and promotes the formation and propagation of amyloid plaques [123].

Soluble CD22 (sCD22) serves as a marker of inflammation and microglial dysfunction [124]. Elevated sCD22 levels have been detected in the peripheral blood of AD patients, are negatively correlated with CSF Aβ42 levels and Aβ42/Aβ40 ratios, and are positively correlated with phosphorylated tau levels and amyloid burden in the brain [125]. Furthermore, elevated plasma sCD22 levels are associated with accelerated cognitive decline, suggesting that sCD22 may accelerate the progression of various neurodegenerative disorders [125].

In the periphery, CD22 regulates B-cell activation and immune responses through interactions with B-cell receptors [126]. The upregulation of CD22 in AD patients may lead to dysregulation of the peripheral immune system, exacerbating inflammation in the CNS. Studies have shown that inhibition of CD22 activity can increase microglial phagocytosis activity and improve clearance of myelin debris, Aβ oligomers, and α-synuclein fibrils, thereby improving cognitive function in murine models [123]. Consequently, CD22 and its associated pathways represent potential therapeutic targets and offer novel strategies for mitigating or halting the progression of various neurodegenerative disorders.

## Conclusions

This review elucidates the importance of bidirectional communication between the nervous and immune systems in AD. We emphasize the role of BBB disruption in facilitating peripheral immune cell infiltration and its role in exacerbating neuroinflammation and AD pathology (Table 1). Specifically, we delineate the roles of various peripheral immune cells, including CD8^+^ T cells, CD4^+^ T cells, and other immune cell populations, in driving AD progression. Furthermore, we summarize the impact of dysregulated key immunological mediators on neurodegeneration. Importantly, these immunological mediators originate not only from peripheral sources but also from within the brain parenchyma itself. The local production of pro-inflammatory factors, APOE4, B2M, and TREM2 in the brain is intimately linked to neurodegeneration, neuroinflammation, and BBB dysfunction. Thus, understanding both the peripheral and central origins of these mediators, and their distinct contributions to AD pathology, is essential for fully deciphering neuroimmune interactions. The meninges - comprising the pia mater, arachnoid mater, and dura mater - protect and encase the CNS while harboring diverse immune cells, including macrophages, dendritic cells, innate lymphoid cells, mast cells, neutrophils, B cells, and T cells [20]. T cell priming occurs primarily in peripheral lymphoid organs, where antigen-presenting cells (APCs), particularly dendritic cells, capture antigens like Aβ and present them to CD4^+^ T cells via MHC-II or CD8^+^ T cells via MHC-I, leading to T cell activation and clonal expansion [127]. In AD, functional meningeal lymphatic vessels are believed to transport CSF-derived molecules and cytokines to the deep cervical lymph nodes (dCLN), enabling interactions between CNS antigens and peripheral T cells; however, this process requires further experimental validation [127–130]. Notably, meningeal lymphatic dysfunction in AD mouse models correlates with increased Aβ deposition in both the meninges and CNS, while enhanced lymphatic function combined with Aβ antibody therapy promotes Aβ clearance [51]. These findings suggest that the meninges not only provide a BBB-independent pathway for macromolecular clearance but also serve as an immune surveillance platform, facilitating interactions between peripheral immune cells and CNS-derived antigens.

**Table 1:** Peripheral immune cell functions and mechanisms in AD

Immune cell type	Main function	Immune factors	References
CD8^+^ T cells	Exacerbate neuroinflammation by secreting cytotoxic factors that activate microglia, leading to neuronal damage and neurodegeneration; increased CD8^+^ TEMRA cells correlate negatively with cognitive function	CCL2, CCL3, CCL4, CCL8, CXCL10, IFN-γ	[5–8, 17, 22]
CD4^+^ T cells - Th1	Activate microglia to promote Aβ clearance; excessive activation exacerbates Aβ accumulation and neuroinflammation, leading to cognitive deficits	IFN-γ, TNF-α	[32–34, 36]
CD4^+^ T cells - Th2	Regulate immune response, reduce inflammation, promote microglial deactivation, alleviating Aβ burden and neuroinflammation	IFN-γ, TNF-α, GM-CSF, IL-2, IL-4	[34, 42]
CD4^+^ T cells - Th9	IL-9, in combination with TGF-β1, promotes differentiation of naive CD4^+^ T cells into Th17 cells	IL-9	[40, 41]
CD4^+^ T cells - Th17	Induce neuroinflammation; inhibit microglial Aβ clearance, accelerating memory impairment and Aβ accumulation	IL-17 A, IFN-γ, IL-23, IL-21, IL-6, TNF-α	[33, 37, 38]
CD4^+^ T cells - Th22	Activates glial cells, promoting pro-inflammatory cytokine production and lymphocyte infiltration into brain parenchyma	IL-22	[39]
CD4^+^ T cells - Tregs	Modulate immune response through secretion of anti-inflammatory cytokines; enhancing Tregs function can be beneficial, while depletion may offer advantages in certain contexts; Tregs are reduced in AD patients	IL-10, TGF-β	[35, 43–48]
B cells	Secrete anti-Aβ immunoglobulins, affecting Aβ accumulation; B cell depletion reduces disease progression, but early depletion accelerates cognitive decline, indicating a complex, stage-dependent role		[71–73]
Macrophages	Involved in Aβ clearance; function declines with age; monocyte-derived macrophages infiltrate brain parenchyma in late-stage AD, potentially compromising BBB integrity	IL-1β, TNF-α	[56, 57, 59–61, 63, 64]
Natural killer cells	Function is complex; depletion provides neuroprotection while augmentation mitigates Aβ deposition and enhances cognitive performance	IL-2, IFN-γ	[65, 66]
Neutrophils	Extravasate and accumulate in amyloid deposition regions, exacerbating AD pathology and contributing to cognitive decline; disrupt BBB integrity	IL-17, NETs	[67–69]
γδT cells	Accumulate in brain and meninges in AD models, associated with cognitive decline; contribute to neuroinflammation and early pathological progression	IL-17 A	[37, 77]
MAIT cells	Elevated MR1 expression in AD brains; promote neuroinflammation and amyloid plaque formation; MR1 or MAIT cell deficiency slows amyloid plaque formation	MR1	[74–76]

Immunosenescence affects T cell priming, resulting in reduced APC function, decreased T cell clonal diversity, and functional impairment [131, 132]. Interestingly, despite the age-related decline in immune responses, AD patients exhibit enhanced T-cell reactivity to Aβ42 peptide [127]. This paradoxical activation likely stems from chronic Aβexposure, persistent low-grade inflammation, and alterations in the immune microenvironment [127, 133]. The heightened T cell response can lead to their abnormal accumulation in the CNS, further exacerbating neuroinflammation and neuronal damage [5, 6, 22].

The role of CD8^+^ T-cells in AD pathogenesis remains controversial. Recent studies using a 3D human neuroimmune axis model demonstrated that CD8^+^ T cells promote neurodegeneration via CXCL10-CXCR3 signaling pathway, leading to enhanced microglial activation [6]. Other work showed that increased CD8^+^ T cells in tau pathological regions correlate with neuronal loss, while inhibiting IFN-γ and PD-1 signaling reduced brain atrophy [22]. Studies using 5×FAD mice yielded conflicting results: one group found CD8^+^ T cell depletion worsened amyloid pathology [134], while another observed these cells accelerated AD-like pathology by targeting disease-associated microglia [29]. These findings suggest context-dependent functions of CD8^+^ T cells in AD, warranting further investigation into their complex roles and mechanisms.

Neurodegenerative diseases such as AD, Parkinson’s disease (PD), Huntington’s disease (HD), and amyotrophic lateral sclerosis (ALS) shared several pathological mechanisms. These include protein misfolding, chronic neuroinflammation, and neuronal loss, which involve interactions between immune cells within and outside the CNS [135–137]. Misfolded proteins like Aβ, tau, and α-synuclein act as danger signals, triggering inflammatory responses through pattern recognition receptors that activate glial cells and promote pro-inflammatory factor release [135, 138, 139]. Immune-related risk genes, such as TREM2 p.R47H variant and HLA-DRB1, highlight the role of immune system dysregulation in these diseases [140–142]. The TREM2 p.R47H variant impairs microglial function and phagocytic capacity, contributing to neuroinflammation in both AD and PD. HLA-DRB1 variations affect antigen presentation and T cell responses, influencing disease progression [140, 141, 143]. The timing of immune responses varies among these diseases: in multiple sclerosis (MS), inflammation precedes neurodegeneration, while in AD, neurodegeneration often triggers inflammatory responses [137, 144–146]. These insights guide the development of immunomodulatory therapies, such as engineering anti-inflammatory macrophages or enhancing regulatory T cell activity to control inflammation while maintaining neuroprotective functions.

Novel therapeutic strategies targeting neuroimmune interactions show significant potential in AD therapeutics. Early intervention strategies using regulatory T cells and targeted antibodies to mitigate immune cell infiltration and neuroinflammation may be crucial in preventing AD progression. Despite these valuable insights, significant gaps in knowledge persist, particularly regarding the stage-specific mechanisms of peripheral immune cells in AD. Future studies investigating the interactions between microglia and peripheral immune cells and their roles in neuroinflammation will be crucial and insightful. Additionally, further characterization and identification of peripheral immune cells or factors as early diagnostic biomarkers for AD may uncover clinically promising prognostic cell types/cell states or molecular markers. Additionally, characterization of key immunological mediators may lead to enhanced precision in targeted therapeutic strategies. Specifically, the effects of APOE4 on BBB integrity and Aβ clearance efficiency, co-aggregation of B2M with Aβ, and the role of TREM2 in regulating microglial function may lead to specific targeting strategies which maximize therapeutic effects while limiting the perturbation of undesired off-target effects.

Understanding these neuroimmune mechanisms is crucial for developing new therapies to prevent AD progression, potentially providing new avenues for clinical intervention.

## Data Availability

Not applicable.

## Abbreviations

AD: Alzheimer’s disease
APC: Antigen-presenting cell
APOE4: Apolipoprotein E4
APP/PS1: Amyloid precursor protein/presenilin 1 (mouse model)
ASO: Antisense oligonucleotides
Aβ: Amyloid-β
ALS: Amyotrophic lateral sclerosis
BAM: Border-associated macrophage
B2M: β2 microglobulin
BBB: Blood-brain barrier
CAA: Cerebral amyloid angiopathy
CCL: Chemokine (C-C motif) ligand
CD: Cluster of differentiation
CNS: Central nervous system
CSF: Cerebrospinal fluid
CXCL: Chemokine (C-X-C motif) ligand
CXCR3: C-X-C motif chemokine receptor 3
dCLN: deep cervical lymph nodes
DS: Down syndrome
EBV: Epstein-Barr virus
EGF: Epidermal growth factor
FOXP3: Forkhead box P3
GM-CSF: Granulocyte-macrophage colony-stimulating factor
GWAS: Genome-wide association study
HLA: Human leukocyte antigen
HD: Huntington’s disease
IFN: Interferon
IFNAR: Interferon α/β receptor
IL: Interleukin
LFA-1: Lymphocyte function-associated antigen 1
LRP1: Low-density lipoprotein receptor-related protein 1
MHC: Major histocompatibility complex
MAIT: Mucosal-associated invariant T
MAPK: Mitogen-activated protein kinase
MDM: Monocyte-derived macrophage
MR1: Major histocompatibility complex class I-related protein
MS: Multiple sclerosis
mTOR: mechanistic target of rapamycin
NET: Neutrophil extracellular trap
NMDA: N-methyl-D-aspartate
NLRP3: NOD-,LRR-,and pyrin domain-containing protein 3
PD: Parkinson’s disease
sCD22: soluble CD22
sTREM2: soluble triggering receptor expressed on myeloid cells 2
STAT: Signal transducer and activator of transcription
TCR: T-cell receptor
TEMRA: T effector memory CD45RA
TGF-β: Transforming growth factor-β
Th: T helper
TNF: Tumor necrosis factor
TREM2: Triggering receptor expressed on myeloid cells 2
VEGFA: Vascular endothelial growth factor A
VLDL: Very low-density lipoprotein
